# Massive Loss of DNA Methylation in Nitrogen-, but Not in Phosphorus-Deficient *Zea mays* Roots Is Poorly Correlated With Gene Expression Differences

**DOI:** 10.3389/fpls.2018.00497

**Published:** 2018-04-19

**Authors:** Svenja Mager, Uwe Ludewig

**Affiliations:** Nutritional Crop Physiology, Institute of Crop Science, University of Hohenheim, Stuttgart, Germany

**Keywords:** epigenetics, DNA methylation, transposable elements, methylome, maize, nitrogen, phosphorus, gene expression

## Abstract

DNA methylation in plants plays a role in transposon silencing, genome stability and gene expression regulation. Environmental factors alter the methylation pattern of DNA and recently nutrient stresses, such as phosphate starvation, were shown to alter DNA methylation. Furthermore, DNA methylation had been frequently addressed in plants with notably small genomes that are poor in transposons. Here, we compare part of the DNA methylome of nitrogen- and phosphorus-deficient maize roots by reduced representation sequencing and analyze their relationship with gene expression under prolonged stresses. Tremendous DNA methylation loss was encountered in maize under nitrogen-deficiency, but much less with phosphorus-deficiency. This occurred only in the symmetrical cytosine context, predominantly in CG context, but also in the CHG context. In contrast to other plants, differential methylation in the more flexible CHH context was essentially absent. In both deficiency conditions a similar number of differentially expressed genes were found and differentially methylated regions (DMRs) were predominantly identified in transposable elements (TEs). A minor fraction of such DMRs was associated with altered gene expression of nearby genes. Interestingly, although these TEs were mostly hypomethylated, they were associated with both up- or down regulated gene expression. Our results suggest a different methylome regulation in maize compared to rice and Arabidopsis upon nutrient deficiencies and point to highly nutrient- and species-specific dynamics of genomic DNA methylation.

**Description of Significance:** DNA methylation suppresses transposons in plant genomes, but was also associated with other genome protective functions and gene expression regulation. Recently it was shown that DNA methylation dynamically responds to several abiotic and biotic environmental factors, but to a large instance, DNA methylation is also heritable. DNA methylation changes have also been reported under phosphorus starvation in rice and Arabidopsis, but its relation with other nutrients and its importance for individual gene expression remains unclear. Here, DNA methylation changes upon the deficiency of two major essential nutrients, nitrogen and phosphorus, were studied in parallel with gene expression responses in maize roots. We show context, nutrient- and species-specific patterns in the methylome, as well as its relation with the nutrient-deficiency transcriptome. While cases of differentially methylated regions in the vicinity of differentially expressed genes were apparent, both positive and negative roles on the gene expression were identified, irrespective of the context.

## Introduction

Plants contain profound adaptation strategies that influence many aspects of growth, development and metabolism, when the two essential macronutrients nitrogen and phosphorus are insufficiently available. Both nutrients are important components of DNA and RNA. Additionally, nitrogen is part of amino acids, proteins, chlorophyll, plant hormones, and secondary metabolites (Yang et al., 2015), while phosphorus is essential for photosynthesis, carbohydrate production, energy provision and serves as a structural element in phospholipids (Wu et al., 2003; Zhang et al., 2014). To cope with nutrient deficiencies, plants have developed a spectrum of adaptation strategies that range from activating more efficient uptake and nutrient utilization to differentially allocating resources. Interestingly, genome wide DNA methylation changes are associated with the adaptation to phosphorus starvation in Arabidopsis and rice (Secco et al., 2015; Yong-Villalobos et al., 2015).

Chromatin packaging is influenced by histone modifications and DNA methylation, thereby producing eu- or heterochromatin and creating easy or blocked access of the transcriptional machinery to the DNA, respectively, and thereby regulating gene expression (Bender, 2002). DNA methylation is potentially involved in the plasticity for adaptation to environmental changes by regulating genome accessibility. DNA methylation in plants is sensitive to environmental conditions and occurs in all possible cytosine (C) contexts, namely the symmetrical CG and CHG contexts, as well as the asymmetrical CHH context, with G being guanine and H being any base but guanine (Pikaard and Mittelsten Scheid, 2014; Secco et al., 2015; Yong-Villalobos et al., 2015). DNA methylation impacts on processes such as pathogen response, genome stability, heterosis, imprinting, regulation of transposable elements (TEs) and gene expression (Vidalis et al., 2016).

Two major mechanistically different categories for DNA methylation can be differentiated: maintenance and *de novo* methylation. Maintenance methylation is a mechanism by which during cell replication the existing methylation positions are directly copied from the parent strand and established in the newly synthesized DNA strand in exactly the same pattern. This is mechanistically straightforward in the symmetric CG and CHG contexts and is accomplished by the maintenance enzyme Methyltransferase1 (MET1) in the CG context and chromomethylase3 (CMT3) in the CHG context (Eichten et al., 2014; Pikaard and Mittelsten Scheid, 2014; Secco et al., 2015). As the CHH context does not provide the methylation information on the template strand during replication, CHH motifs require *de novo* RNA-directed DNA methylation (RdDM) after replication. Though RdDM occurs in all contexts, it is most prominent in the CHH context, where RNA polymerase IV produces single-stranded RNA transcripts that are subsequently converted to double-stranded RNAs by RNA-dependent RNA polymerase 2 (RDR2) and then processed to 24-nucleotide small interfering RNAs (siRNAs) by Dicers. These are loaded onto Argonaute 4 (AGO4) and guided to RNA polymerase V-transcribed RNA scaffolds. Finally, Domains 10 Rearranged Methyltransferase 2 (DRM2) is recruited to place *de novo* methylations (Lister et al., 2009; Dowen et al., 2012; Matzke and Mosher, 2014; Secco et al., 2015). Loss of DNA methylation, by contrast, may happen passively, in case of a lack of maintenance methylation during replication or DNA repair. Furthermore, active loss of 5-methylcytosine happens through Repressor of silencing 1 (ROS1) and Demeter (DME) proteins. These contain DNA glycosylase domains for base excision repair (Eichten et al., 2014; Pikaard and Mittelsten Scheid, 2014; Park et al., 2017).

DNA methylation patterns were recently reported to profoundly be affected in phosphorus starvation, with major methylation resetting in rice that occurred after correlated gene expression changes (Secco et al., 2015). In *Arabidopsis thaliana*, global methylation differences were also observed under phosphorus starvation that was to some extent correlated with phosphate-starvation-induced gene expression differences (Secco et al., 2015; Yong-Villalobos et al., 2015). Interestingly, TE hypermethylation (that is associated with their blocking) correlated with increased expression of nearby genes in rice (Secco et al., 2015). Furthermore, high DNA methylation in promoters or near transcription start sites (TSS) often shuts down expression, but there are also examples where gene body methylation stabilizes expression (Li et al., 2015; Suzuki and Bird, 2008).

The understanding of the interaction of DNA methylation with environmental stresses, especially nutrient stresses and whether DNA methylation patterns can be inherited, is still in its infancy. Furthermore, the relevance of transposon silencing in *Zea mays*, with its 2.3 gigabase genome (18-fold larger than Arabidopsis and comprising 85% vs. 10% TEs) appears of much larger magnitude in crops (Arabidopsis Genome Initiative, 2000; Feschotte et al., 2002; Zhang and Wessler, 2004; Schnable et al., 2009; Tenaillon et al., 2011). Therefore, the influences of nitrogen- and phosphorus-deficiency on the *Z. mays* root transcriptomes and methylomes were studied in the maize inbred line B73. We hypothesized that the methylome of maize roots is well correlated with gene expression changes upon specific nutrient deficiencies. As the large, repetitive genome of maize impedes simple deep coverage of the DNA methylation pattern, we used reduced representation bisulfite sequencing (RRBS) (Li et al., 2014) to monitor methylation in a defined subset of the genome. We identified cytosine-context specific major methylation loss under nitrogen-deficiency and defined differential methylation in phosphorus-deficient roots. Parallel RNA-sequencing to the same maize root samples allowed the identification of expression changes in genes located near differentially methylated TEs, but both up- and down-regulation of genes was observed with differential methylation.

## Materials and Methods

### Plant Growth Conditions

The *Z. mays* inbred line B73 plants were grown in hydroponic culture under controlled conditions in a climate chamber with simulated day length of 16 h at 25°C and 8 h night length at 20°C. Humidity amounted to 60–80% and photosynthetically active photon flux density (PFD) was 400 μmol m^-2^ s^-1^. Maize seeds were surface-sterilized by rinsing them for 2 min in a 10% H_2_O_2_ solution. The seeds then stayed in a 10 mM CaSO_4_ solution for 24 h and were then laid between foam sheets soaked in a 3 mM CaSO_4_ solution for 4 days to germinate. The seedlings were put for 3 days into pots (6 plants each) containing 2.8 L of a diluted maize nutrient solution with 0.1 mM K_2_SO_4_, 0.12 mM MgCl_2_, 0.5 mM Ca(NO_3_)_2_ and 200 μM KH_2_PO_4_. Micronutrients were added at the following concentrations: 0.2 μM H_3_BO_3_, 0.1 μM MnSO_4_, 0.1 μM ZnSO_4_, 0.04 μM CuSO_4_, and 2 nM (NH_4_)_6_Mo_7_O_24_. After 3 days, the seedlings were separated into two seedlings per pot and the nutrient concentrations in the solution were increased to: 0.5 mM K_2_SO_4_, 0.6 mM, MgCl_2_, 2.5 mM Ca(NO_3_)_2_ and 0.1 mM KH_2_PO_4_. The KH_2_PO_4_ concentration was progressively raised to 0.2 mM in the 3rd week and to 0.5 mM in the 4th week after starting the treatment. Micronutrients were supplied as: 1 μM of H_3_BO_3_, 0.5 μM MnSO_4_, 0.5 μM ZnSO_4_, 0.2 μM CuSO_4_, 0.01 μM (NH_4_)_6_Mo_7_O_24_, 100 μM Fe-Sequestrene, which was raised to 200 μM at the first nutrient solution change and to its final amount of 300 μM at the second solution change. The deficiency treatments started after 1 week, the nitrogen deficiency plants then obtained 90 μM of Ca(NO_3_)_2_ and phosphorus deficient plant received 18 μM KH_2_PO_4_. The first nutrient solution change was done after 7 days and from then on every 3 days till the harvest at 5 weeks after germination (4 weeks after treatment start).

### Sample Taking and Nutrient Analysis

For the nutrient analysis, the second and third youngest leaves were taken from each plant. For RRBS and RNA-sequencing, root material was taken. For each analysis, two plants from one pot were pooled, meaning that for each treatment (and for the control) three replicates with two plants per replicate were used. For the nutrient analysis, the leaf material was measured for nitrogen and phosphorus content. For both nutrients, plant material was digested via microwave (VDLUFA, 2011), the phosphorus was subsequently measured via UV-VIS spectroscopy and nitrogen after Kjeldahl (1883). Data are given as means ± SD.

### DNA Extraction and Reduced Representation Bisulfite Sequencing

DNA was extracted from root material with three replicates per condition (Control, -N and -P), with each replicate including material from 2 plants, resulting in nine samples. DNA was extracted from the root material according to manufacturer’s protocol of the Qiagen DNeasy Plant Mini Kit. If necessary, the DNA was concentrated and cleaned via alcohol precipitation. Quality and quantity of the DNA were verified with the Thermo Scientific Nanodrop 2000c Spectrophotometer and via Qubit Fluorometric Quantitation. DNA samples all had OD260/280 ≥ 1.8. Reduced representation bisulfite sequencing (RRBS) was used to monitor methylation (Li et al., 2014), in which a partial high density coverage of the genome methylation profile allows a representative genomic view (Smith et al., 2009; Martinez-Arguelles et al., 2014). The DNA was digested via the restriction enzyme MspI, specific for a CG-containing motif, and size-selected for sequences being 40–220 bp in length to produce a reduced representation genome (RRG). Restriction digest and size-selection was done by Beijing Genomics Institute (BGI, China) as well as the reduced representation bisulfite sequencing, which included library construction and 100 bp paired-end sequencing on the Illumina Hiseq2000.

### Processing and Analysis of RRBS Data

Clean data provided by BGI was checked with FastQC (Babraham Bioinformatics). The first 4 and the last 6 basepairs of each read were then removed via the FastX-Toolkit by Hannon Lab to remove bias and get higher quality reads. As reference the *Z. mays* genome (AGPv3) provided by the Maize Genetics and Genomics Database (Sen et al., 2009) was used. The alignment was done by BS-Seeker2, which produces a reduced representation genome from the reference genome to increase mappability and accuracy of the alignment by virtually cutting with MspI and size-selecting for sequences of 20–400 bp length (Guo et al., 2013). The broader range during virtual size-selection of the reference genome compared to size-selection for libraries of the samples was chosen because size-selection of digested DNA samples often is not perfectly precise and therefore might include slightly smaller or longer sequences than intended. Alignment was done using the reduced representation reference genome and with default settings except for using bowtie2 (Langmead and Salzberg, 2013) instead of bowtie as short read mapper. After alignment, BS-Seeker2 was used to call methylation levels from the mapping results using the default settings.

The methylation information was then used in DMRcaller (Zabet and Tsang, 2015) provided by Bioconductor to determine differentially methylated regions (DMRs) and visualize methylation level distribution over whole chromosomes. The methylation levels determined by BS-Seeker2 for each replicate were pooled together by DMRcaller when loading in the data, so that the methylation level values of all three replicates of one condition were stored in a GRanges object. The program used an algorithm including smoothing (depicted as noise_filter with “triangular” kernel in DMRcaller) described by Hebestreit et al. (2013) to compute differentially methylated cytosines. For determination of DMRs the score test, specified by DMRcaller, was exerted. DMRs were defined as follows: Minimum size of 50 bp with a window size of 500 bp, a minimum number of 4 cytosines per DMR, a minimum proportion difference of methylation of 40%, as well as a *p*-value below 0.01. A list of TEs (ZmB73v3) provided by Unité de Recherche Génomique Info (Jamilloux et al., 2017) was used to define which DMRs were located in TEs. To specify DMRs located in promoters, the region 2000 bp upstream of each gene was arbitrarily set as promoter region. DMRs in genes were determined with gene information taken from maize annotation files (AGPv3) provided by the Maize Genetics and Genomics Database (Sen et al., 2009).

### RNA Extraction and RNA Sequencing

RNA samples were taken from the same roots as the DNA samples. Total RNA was extracted according to manufacturer’s manual with the analytik jena innuPREP Plant RNA Kit. Quality and quantity were examined via Thermo Scientific Nanodrop 2000c spectrophotometer and the Agilent 2100 Bioanalyzer. As for the DNA samples, only RNA samples with OD260/280 ≥ 1.8 were used. Truseq 160 bp short-insert library construction as well as 100 bp paired-end sequencing on Hiseq 4000 was done by Beijing Genomics Institute (BGI, China).

### Processing and Analysis of RNA-Seq Data

Clean data was examined via FastQC (Babraham Bioinformatics) and aligned with HISAT2 (Kim et al., 2015) against the same *Z. mays* reference genome as was used for RRBS. Alignment was done mainly with options and the additional options –phred64, –dta-cufflinks, –no-mixed, and –no-discordant. Transcript assembling was done via cufflinks from the cufflinks suite of tools (Trapnell et al., 2010) with the –GTF-guide (using the same annotation files as for RRBS) and –no-effective-length-correction and otherwise default options. Afterwards assemblies were merged with cuffmerge using the -g and the -s options. Finally, with cuffdiff the significant differentially expressed genes were determined. As options, -compatible-hits-norm, -b and -u were used.

### Correlating DNA Methylation and Gene Expression

To determine if DMRs in gene body or promoter region influence gene expression of that gene, Fisher’s exact test on a 2 × 2 contingency table was applied. A 5% significance level was used. The contingency table contained the number of genes differentially methylated and expressed, number of genes only differentially methylated, number of genes only differentially expressed and number of genes neither differentially expressed nor methylated. However, for this test only genes were taken into account that were covered by the RRG with at least 500 bases. This cutoff was set to avoid comparing a lot of genes for which no methylation information but only expression information was available. This reduced false negative results (by assuming that no DMR is present in a gene for which no methylation information was present). A minimum of 500 covered bases was chosen as a compromise between losing too many genes with DMRs and keeping too many genes without methylation information.

For investigation of whether differential methylation in TEs influences the expression of the closest gene, we first used BEDTools to determine the closest gene for each TE regardless of being upstream or downstream of the TE (Quinlan and Hall, 2010). Only those TEs were taken into account, which were covered by the reduced representation genome at all. This difference in setting the cutoff for genes and TEs was done because many TEs are very short und setting another cutoff lost a lot of TEs. Again, via Fisher’s exact test with a significance level of 5% on a 2 × 2 contingency table, we then determined the dependence of differential methylation in TEs and differential expression of the closest gene. For determination of linear correlation between differentially methylated genome features and gene expression, scatter plots were done with methylation proportion difference against the log_2_-fold change of expression.

## Results

### Hydroponic Growth and Nutrient Deficiency Conditions

**FIGURE 1:**
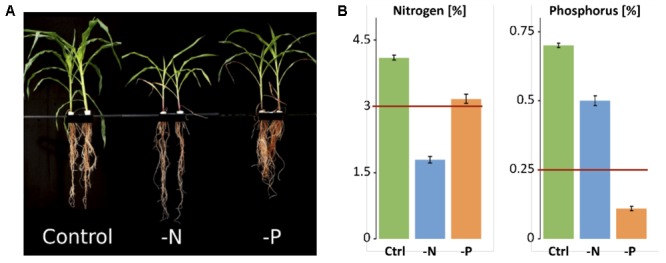
Phenotype and nutrient content of control and deficiency samples. **(A)** Hydroponically grown maize plants harvested at age of 5 weeks. **(B)** Nitrogen and phosphorus concentration in maize shoots. Minimum amount needed by maize is indicated by red line.

Maize B73 lines were grown in hydroponic culture in growth chambers. Control plants showed vigorous growth, while plants receiving 28-fold less nitrogen or 6-fold less phosphorus showed the typical deficiency symptoms: For nitrogen-deficiency, restricted shoot growth, pale green leaf color due to decreased photosynthesis and chlorosis in older leaves were observed (Boussadia et al., 2010; Comadira et al., 2015). Likewise, reduced shoot and more complex root growth, resulting in increased root-to-shoot biomass ratio and dark green leaves with anthocyanin accumulation were observed in -P plants. The red color from anthocyanin accumulation was most prominent in the stems. These visual symptoms were indicative of typical phosphorus-deficiency (**Figure 1A**). While control shoots contained 4.1% N and 0.7% P in their dry mass, -N plants contained only 1.79% N and 0.5% P (**Figure 1B**). -P plants contained 3.17% N and 0.11% P, confirming the specific, severe systemic nutrient deficiencies in the samples that are typically seen below 3% for N and below 0.25% for P in young maize (Sahrawat, 2014).

### Reduced Representation Bisulfite Sequencing and Nutrient-Specific Methylomes

**FIGURE 2:**
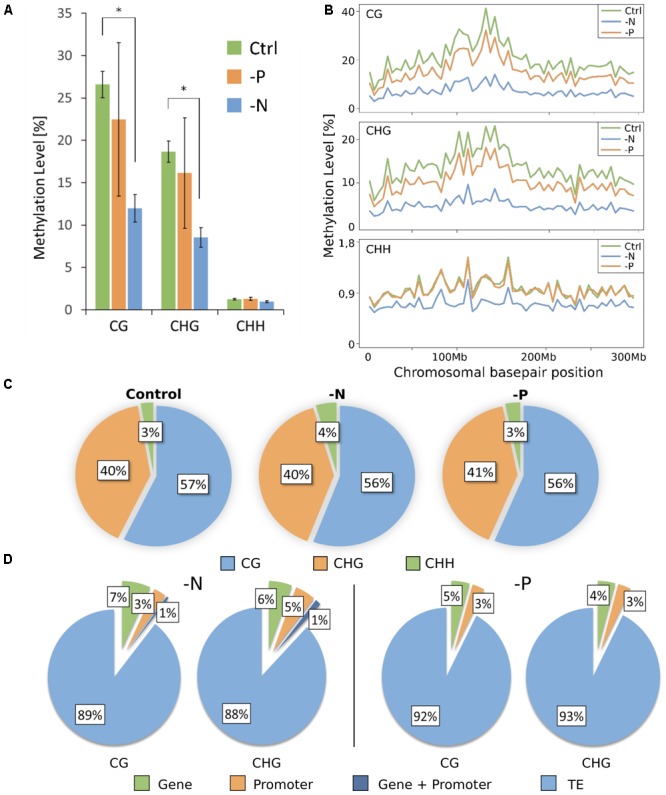
Genome-wide DNA methylation pattern. **(A)** DNA methylation level (means ± SD) depends on cytosine sequence context. **(B)** Methylation profile across chromosome 1. Each point corresponds to an average methylation across 5 million base pairs. **(C)** Proportion of each context contributing to the total amount of methylated cytosines. **(D)** Percentage share of DMRs present in different genome features. TE, transposable element.

The reduced representation genome methylation profile of roots was analyzed in triplicate for each treatment, from pooled plant samples. Bisulfite conversion rate was always >98%. Virtual digestion of the maize genome with MspI and subsequent size-selection revealed that about 14% of the real genome was covered by the reduced representation genome. For all samples, independent of the cytosine context, 90% of all cytosines were covered by at least 5 reads (Supplementary Figure S1). Mapping ability for all samples was >48% (Supplementary Table S1). Overall, RRBS processing showed sufficiently high coverage and mappability for reliable downstream analyses. Across the reduced representation genome (RRG), a massive loss of methylation in CG and CHG contexts for -N was measured. In control, 26.6% of all cytosines in the CG context were methylated, whereas in -N samples, the average methylation level in the CG context was significantly less, only 12.0% (**Figure 2A**). Minor overall methylation loss in -P was also observed, a mean reduction to 22.5% of CG methylation. However, a large variance of the methylation was encountered within the three sequenced -P samples, where the total methylation amount (averaged over the entire genome) ranged from 12.6 to 30.4%. The same trend was found in the CHG context, where 18.7% were methylated in the control samples, 16.2% in -P samples and only 8.5% in -N. By contrast, the CHH context was almost unaffected by the deficiencies, although CHH methylation was present. Cytosines in this context were the least methylated for all samples and this methylation in -N samples was also lowered, from 1.26% in control to 0.96%, while methylation in -P was even slightly larger than under control conditions (1.29%, **Figure 2A**). Whether the methylation changes were distributed uniformly across chromosomes was analyzed by low-resolution profiles of average methylation levels in a grid of 5 million bases via DMRcaller (**Figure 2B**). As a representative, chromosome 1 methylation is shown for the three different cytosine contexts and control, -N and -P conditions. As for the general methylation levels, a rather uniform reduction of CG and CHG methylation was measured, maintaining the higher overall methylation level in centromeric regions. In the CHH context, by contrast, -P was almost exactly the same as the control and the methylation reduction in -N was less pronounced than in the other two contexts. In the CHH context, the larger methylation in the centromeric region was less pronounced (**Figure 2B**). The relative contribution of each context to the total number of methylated cytosines was, however, unaffected by the deficiencies (**Figure 2C**).

As isolated, individual base methylation changes appear to have little functional relevance, only strongly DMRs were considered. DMRs were defined as regions of 50 to 500 bp in length, containing at least 4 cytosines and 4 reads per cytosine, which differ by more than 40% in methylation, with *p*-value of ≤0.01. In agreement with the massive loss of methylation in -N and minor methylation losses in -P, more DMRs were determined for -N. Most DMRs were present in the CG context and the majority of these were hypomethylated, but not a single DMR was encountered in the CHH context (**Table 1**). With less strict DMR criteria (minimum of three cytosines with at least three reads per cytosine and a minimal methylation difference of only 10%) only 6 DMRs for -N and 2 for -P were identified in the CHH context (Supplementary Table S2), whereas CG and CHG DMR numbers increased between 2.8- and 7.6-fold for -N and -P samples. Still CHH DMRs were negligibly small, so we decided to stick to stricter DMR criteria to consider most severely affected chromosomal regions, which in previous studies were associated with substantial transcriptional differences (Secco et al., 2015).

**Table 1:** Differentially methylated regions (DMR) count (≥40% methylation difference).

	-N	-P
DMRs CG	1655 (97.7%)	461 (77.2%)
DMRs CHG	170 (92.4%)	90 (75.6%)
DMRs CHH	0	0

*Percentage of hypomethylation in parenthesis*.

In both contexts, DMRs were most pronounced in TEs. 89 and 88% of all DMRs in CG and CHG contexts, respectively, fell into that class in -N and 92 and 93% for CG and CHG contexts, respectively, in -P (**Figure 2D**). Taking into account that transposons make up roughly 85% of the maize genome, DMRs were slightly overrepresented in these regions. DMRs that stretched across both promoter and gene bodies were rare, with 1% in both -N contexts and none in -P. DMRs in genes were slightly more abundant in -N with 6–7%, while 4–5% of DMRs were in genes in -P. DMRs in promoters amounted to 3% in all cases, except for the CHG context in -N, where it was 5% (**Figure 2D**).

### The Maize Root Transcriptome Under Prolonged Nutrient-Deficiency

**FIGURE 3:**
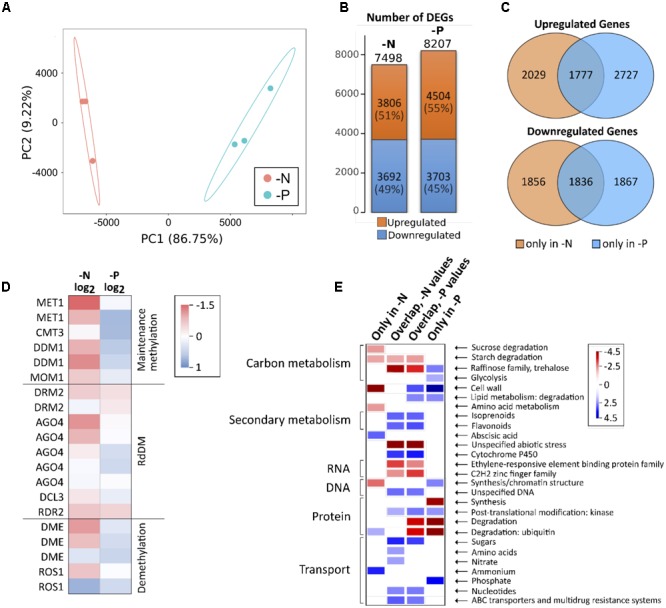
Gene expression in -N and -P samples. **(A)** PCA of normalized FPKM expression data for -N and -P samples. **(B)** Number of up- and downregulated DEGs. DEG, differentially expressed gene. **(C)** Venn diagrams showing the number of DEGs being up- or downregulated in -N or -P or in both. **(D)** Color code for the expression of enzymes involved in DNA methylation or demethylation. **(E)** Selected categories of functional ontologies for expression data. Shown are genes which are only differentially expressed in -N or -P as well as genes which are differentially expressed in both treatments (overlapping DEGs).

N and P nutrient deficiencies rapidly alter gene expression in roots, but some initially strongly regulated genes abate to initial levels after some days, while a minority persists being different, often associated with developmental and metabolic changes under deficiency (Secco et al., 2015; Menz et al., 2016). We aimed to capture the transcriptomic differences compared to the control after prolonged nutrient deficiency, at the same time point when the methylome analyses were made. The alignment rate for control, -N and -P samples to the reference genome was quite similar being 90% for -P and 91% for control and -N (Supplementary Table S3). After alignment, transcripts were assembled and all differentially expressed genes were determined. A principle component analysis (PCA) gave a first impression about variation between samples and the variation between the replicates within samples. It separated the datasets into two groups, in accordance with the distinct deficiencies. The principle component 1 (PC1) (**Figure 3A**) reflected differences in the gene expression between -N and -P and accounted for the majority of the variance (almost 87%) in the data. A smaller part of the variance (9%) was explained by PC2 (**Figure 3A**), which depicts the variability between replicates. Hence, the PCA1 accounts for the well-fractionated gene expression differences according to the two deficiency treatments and suggests homogeneity between samples. The total number of significantly differentially expressed genes (DEGs) was similar for -P and -N samples, with -N having 7498 DEGs and -P 8208 DEGs of a total of 39469 identified *Z. mays* genes in roots (**Figure 3B**). Similar numbers of up- and down-regulated DEGs were detected, with 49% down-regulated DEGs in -N and 45% in -P. About 3600 genes were differentially regulated in both -N and -P, of which about half were collectively down- and half were up-regulated in both treatments (**Figure 3C**). In addition, little less than 500 genes were differentially expressed in both treatments, but in opposite directions (not shown).

As CG methylation was massively lost in -N, we hypothesized that genes coding for maintenance methylation enzymes as well as enzymes involved in RNA-directed DNA methylation were down-regulated in -N. In addition, demethylating enzymes might be up-regulated. These hypotheses were largely substantiated by the RNA-seq data, although all methylation-related genes were expressed at relatively low levels in the roots (**Figure 3D**). The majority of enzymes involved in methylation was either unchanged or down-regulated in -N, while these genes were weakly up-regulated or unchanged in -P. For demethylating enzymes, the results were less consistent in -N, where DME and ROS1 enzymes were both up- or down-regulated. By contrast, the coding genes for demethylating enzymes were slightly higher expressed in -P, as were the opposing genes involved in methylation maintenance (**Figure 3D**).

The gene expression in -P and -N verified that gene categories commonly associated with these particular deficiencies were overrepresented in the differentially expressed genes. **Figure 3E** summarizes selected differentially regulated gene categories. We split these differentially expressed genes into those that were exclusively differentially expressed under -N, those selectively appearing in the -P condition and finally those that were associated with both deficiencies, -N and -P. In -N, the cell wall, sucrose, starch and amino acid metabolism was collectively down, while protein degradation via the ubiquitin pathway, abscisic acid signaling and ammonium uptake were collectively up-regulated categories. By contrast, in -P, selective sugar pathways, glycolysis, cell wall metabolism, kinases, lipid degradation and phosphate uptake transport were up-regulated, while protein synthesis and degradation were repressed. Interestingly, the large number of co-regulated genes in -N and -P identified categories that likely represent more unspecific and secondary stress, as expected from the prolonged growth under deficiency conditions. This is supported by the finding that under these conditions, carbon metabolism was repressed, but secondary metabolism was up-regulated. Furthermore, categories associated with stress responses were differentially regulated and sugar, nucleotide and multidrug resistance transport was up. More specifically, amino acid and nitrate transport up-regulation was only seen in the -N samples, in agreement with our expectations (**Figure 3E**).

Furthermore, within the -N samples, crucial N deficiency-regulated marker genes were found differentially expressed. For example, genes encoding high affinity nitrate uptake systems (nitrate transporter 2 class), as well as high affinity glutamate-ammonia ligases (=glutamine synthetases) were substantially up-regulated (**Table 2**). By contrast, three genes encoding nitrate reductase were massively down in -N, in agreement with their common strong nitrate-induced gene expression (Schluter et al., 2012). Likewise, key high affinity phosphorus-uptake related genes were up-regulated in -P, namely the genes for inorganic phosphate transmembrane transporters 1;4 (PHT1;4) and phosphatases, of which some may even be released from roots for mobilizing organic P from the soil (**Table 3**). Furthermore, genes encoding SPX domains, which are components of many proteins involved in phosphate transport and signaling (Wild et al., 2016), were strongly up-regulated. SPX domains seem to help sensing limited P amounts and are involved in the P starvation responses (Duan et al., 2008; Secco et al., 2013; Wild et al., 2016). Additionally, genes encoding proteins needed for phosphorus-independent bypass glycolysis reactions were up-regulated, like phosphoenolpyruvate carboxylase (PEPC) and sucrose-phosphate synthase (SPS). Finally, many genes encoding proteins involved in lipid homeostasis and metabolism, tentatively in readjusting membrane lipids to potentially reduced phospholipid levels, were also up-regulated, among them being Lipase class 3 family proteins, UDP-sulfoquinovose:DAG sulfoquinovosyltransferase and UDP-galactosyltransferase (**Table 3**).

**Table 2:** Nitrogen-deficiency regulated differentially expressed genes.

Gene ID	Annotation	FPKM Ctrl	FPKM -N	Log_2_ FC
GRMZM2G010280	Nitrate transporter 2:1	170.3	674.5	1.99
GRMZM2G010251	Nitrate transporter 2:1	115.2	244.5	1.09
GRMZM2G455124	Nitrate transporter 2:5	3.6	349.2	6.60
GRMZM5G878558	Nitrate reductase 1	251.0	1.5	–7.35
GRMZM2G568636	Nitrate reductase 1	167.8	45.2	–1.89
GRMZM2G102959	Nitrate reductase 1	243.3	1.9	–7.03
GRMZM2G036464	Glutamate-ammonia ligase	121.9	680.1	2.48
GRMZM5G872068	Glutamate-ammonia ligase	240.5	660.8	1.46

**Table 3:** Phosphorus-deficiency-induced differential gene expression.

Gene ID	Annotation	FPKM Ctrl	FPKM -N	Log_2_ FC
GRMZM2G326707	Inorganic phosphate transmembrane transporter, PHT1;4	73.0	305.5	2.07
GRMZM2G154090	Inorganic phosphate transmembrane transporter, PHT1;4	6.7	695.6	6.69
GRMZM2G112377	Inorganic phosphate transmembrane transporter, PHT1;4	1.4	271.3	7.64
GRMZM2G069542	Phosphoenolpyruvate carboxylase	92.7	154.5	0.74
GRMZM2G074122	Phosphoenolpyruvate carboxylase	42.7	73.6	0.79
GRMZM2G110714	Phosphoenolpyruvate carboxylase	3.2	22.9	2.83
GRMZM2G008507	Sucrose-phosphate synthase	1.2	77.9	6.02
GRMZM2G047995	Lipase class 3 family protein	4.0	87.0	4.45
GRMZM2G169562	Lipase class 3 family protein	2.9	27.3	3.23
GRMZM5G829946	Senescence-related gene 3, glycerophosphodiester phosphodiesterase	0.3	159.3	8.92
GRMZM2G064962	Glycerophosphoryl diester phosphodiester family protein	19.2	108.0	2.49
GRMZM2G315848	Protein nucleotide pyrophosphatase/phosphodiesterase	6.7	65.8	3.29
GRMZM2G477503	Sulfoquinovosyldiacylglycerol 2	6.5	348.0	5.75
GRMZM2G141320	1,2-diacylglycerol 3-beta-galactosyltransferase/UDP-galactosyltransferase	0	159.8	–
GRMZM2G152447	Acid phosphatase/protein serine/threonine phosphatase	1.0	318.8	8.34
GRMZM2G138756	Acid phosphatase/protein serine/threonine phosphatase	0.1	17.7	8.00
GRMZM5G836174	Phosphatase	0.5	1991.8	11.83
GRMZM2G015908	Phosphatase	3.9	309.9	6.30
GRMZM2G021106	Phosphatase	0.4	48.1	6.81
GRMZM2G171423	SPX domain gene 2	1.0	84.6	6.46
GRMZM5G805389	SPX domain gene 3	3.3	1246.0	8.57
GRMZM2G065989	SPX domain gene 3	2.5	1008.0	8.65
GRMZM5G828488	SPX domain gene 3	0.5	399.5	9.65

### Correlation of DNA Methylation With Transcriptional Changes

**FIGURE 4:**
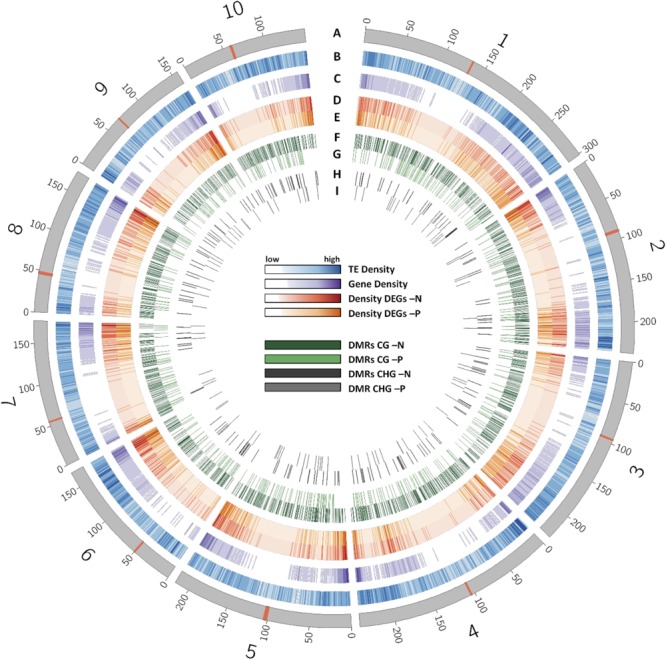
Distribution of different features across the chromosomes of the B73 genome. **(A)** Chromosomes with their centromere shown as red band. **(B)** Density of transposable elements. **(C)** Density of genes. **(D)** Density of DEGs in -N. **(E)** Density of DEGs in -P samples. **(F)** Distribution of CG DMRs in -N samples. **(G)** Distribution of CG DMRs in -P samples. **(H)** Distribution of CHG DMRs in -N samples. **(I)** Distribution of CHG DMRs in -P samples.

Reduced representation bisulfite sequencing and RNA-sequencing results were then used to identify possible correlations between methylation, nutrient-deficiencies and accompanying gene transcriptional changes. The entire chromosomal distribution of TEs, genes, DEGs and DMRs in the whole maize genome with its 10 chromosomes is shown in **Figure 4**. Centromeric regions (Wolfgruber et al., 2009) are indicated as red bands. TEs were relatively equally distributed over each chromosome, while the gene density was clearly enriched toward the terminal ends of each chromosome arm. Gene density was depleted in centromeres and centromere-flanking regions. In agreement with the higher gene density at the outer chromosomal ends, DEGs in -N and -P were enriched in these regions. In contrast to that, CG and CHG DMRs were relatively uniformly distributed across each chromosome, both for -N and -P. The large overlap of TEs with DMRs is consistent with the fact that most DMRs were positioned in TEs.

**FIGURE 5:**
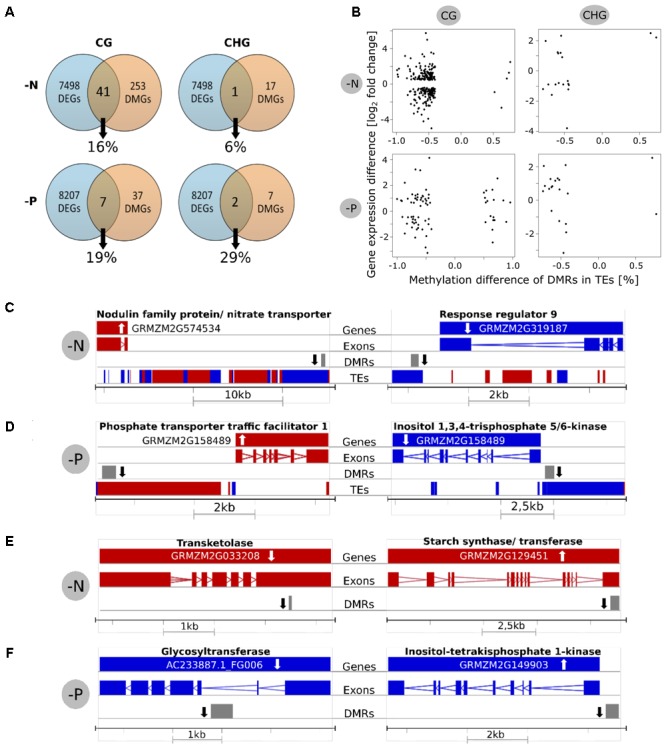
Relationship between differential methylation and gene expression. **(A)** Venn diagrams showing how many differentially methylated genes (DMGs) were also differentially expressed genes (DEGs). Percentages are relative to DMGs. **(B)** Scatterplots for investigation of linear correlations between methylation and expression. The *x*-axis shows the methylation difference of DMRs in TEs and the *y*-axis shows the gene expression of the closest gene. **(C,D)** Examples for differentially methylated TEs in CG context and altered expression of the closest gene. **(E,F)** Examples for differentially methylated genes in CG context with differential expression. White arrow = Gene expression up/down, black arrow = methylation up/down, red = forward strand, blue = reverse strand.

As differential methylation in a gene or its promoter region might influence the expression of this gene and vice versa, we determined the percentage of differentially expressed genes with DMRs in their gene body or promoter (**Figure 5A**). As mentioned above, because reduced representation methylome sequencing was used, only roughly 14% of the entire genome sequence was covered. However, all transcripts were considered. Keeping this limitation in mind, 41 of a total of 253 differentially methylated genes in the CG context were differentially expressed in -N, being 16% of the genes with DMRs. In -P, 7 out of only 37 differentially methylated genes were also differentially expressed, accounting for 19%. In the CHG context, only one gene (-N, 6%) or two genes (-P, 29%) were differentially methylated and expressed at the same time.

For the examination of the statistical significance of these observations, we further quantified the correlation of DEGs and close DMRs. We took only genes into account that were covered by the reduced representation methylome by at least 500 base pairs. For the analysis of differentially methylated TEs and their potential influence on the expression of the closest gene, however, no such cutoff was applied, as many TEs were very short and otherwise escaped from the analysis. A significant correlation (at 5% level) of methylation and gene expression was suggested by Fisher’s exact test only for CG methylation in TEs next to DEGs (*p*-value 0.003 for -N and 0.009 for -P; **Table 4**). However, a lack of significant correlations between differentially methylated genes or promoters in any context and differential gene expression was encountered.

**Table 4:** *P*-values of Fisher’s exact test on dependence of differential methylation in genes, promotors or TEs and gene expression.

	-N		-P	
	CG	CHG	CG	CHG
DMR in genes	0.059	0.220	0.440	0.552
DMR in promotors	0.579	1.000	0.231	0.414
DMR in TE	0.003	0.594	0.009	0.666

These findings were validated by scatter plots showing differential methylation in TEs vs. gene expression of the closest genes (**Figure 5B**). The predominant reduced methylation was frequently involved in up- or down-regulated gene expression, without preference, irrespective for -N or -P. The same holds true for the few hypermethylated TEs, mostly found in the -P CG context. A similar plot for methylated genes/promoters vs. their gene expression difference also identified a similar scattered appearance (Supplementary Figure S2). However, neither for hypomethylation, nor for hypermethylation, clear trends of gene repression or gene activation were apparent. The same holds true in plots with the expression of all genes (not only significantly differentially expressed ones) and the DMRs in TEs or genes (Supplementary Figure S3).

As hypomethylation of TEs may induce their expression, we also checked for expression of TEs in our dataset. Within all detected transcripts, only 484 sequences were annotated to contain TE sequences. However, the number of expressed TEs did not change in the deficiencies, while the average expression level of these transcripts was moderately increased by 23% in -N, and by 20% in -P, compared to the control.

Among the genes that were differentially expressed and differential methylated in the gene body, promoter or a nearby TE, several were among nutrient-specific deficiency-regulated genes. The position of the DEGs, together with their gene structure is shown for few examples in **Figures 5C–F**. This includes a gene annotated as a putative induced nitrate transporter close to a hypomethylated transposable upstream element in -N. By contrast, a hypomethylated TE was close to down-regulated gene of response regulator 9 in -N (**Figure 5C**). Similar cases are shown for -P, where hypomethylation in -P up- or down regulated P-related genes, respectively (**Figure 5D**). Hypomethylation in a neighboring TE was associated with up-regulation of phosphate transporter traffic facilitator 1, while inositol 1,3,4-trisphosphate 5/6-kinase was less expressed next to a hypomethylated TE. Examples for genes that were both differentially methylated in or close to the gene body (but not in transposon sequences) and that were differentially expressed are shown in **Figures 5E,F**. A down regulated transketolase and an up-regulated starch synthase were both hypomethylated in -N. A hypomethylated glycosyltransferase was decreased in expression, while a hypomethylated inositol-tetrakisphosphate 1-kinase was higher expressed in -P. Taken together, only a minor correlation between differentially methylated TEs and the expression of closely neighbored genes was observed, while gene expression and direct methylation were in most cases remarkably independent of each other.

## Discussion

Previous research indicated that especially phosphorus deficiency strongly affected DNA methylation, but results from rice and Arabidopsis did not yet reveal a common mechanistic understanding of the underlying mechanisms. Here, we compared the methylome and transcriptome of *Z. mays roots* grown under either nitrogen- or phosphorus-deficient conditions. Growing the plants in a controlled environment allowed us to investigate parallel methylation and transcriptional changes caused by a single nutrient deficiency, ruling out other environmental impacts. Phenotypic analysis of the plants as well as the nutrient analysis confirmed that the plants were specifically stressed from lack of the intended nutrient. Furthermore, the induction of typical starvation-induced genes confirmed the induction of a certain nutrient deficiency. We focused on root tissues for analysis of methylome and transcriptome, as this is the most important plant organ for nutrient sensing and take-up. Previous research suggested that most plant tissues do not vary tremendously in their DNA methylation (Roessler et al., 2016), but there are also massive changes reported between organs (Seymour et al., 2014). One of our main interests was to find out if methylation adapts not only to environmental stresses, like lack of nutrients, in a general stress-related way, but also specifically depends on which nutrient is lacking. Maize root samples from plants grown in nitrogen deficiency experienced an immense loss of methylated cytosines, especially in the symmetrical contexts, while phosphorus-deficiency samples showed only a much smaller loss in the symmetrical contexts, providing evidence for a nutrient-specific adaptation in DNA methylation. However, methylation in the asymmetrical context did not change.

As CHH methylation is placed *de novo* on the DNA and thereby believed to be more and faster adaptable to environmental conditions than CHG and CG contexts, we assumed that CHH DNA methylation would vary the most between control and deficiency plants. Interestingly, the most flexible context was CG, while the CHH context was less methylated in general. A negligible number of DMRs were found in the CHH context, even with relaxed criteria for defining a DMR. These findings are in accordance with Eichten et al. (2013) and Li et al. (2015), who also recognized low methylation in the CHH context and only a very small number of DMRs compared to the symmetric contexts in different maize inbred lines, including B73. The changes in DNA methylation were present after 4 weeks of treatment, indicating long-term changes, though it is not clear whether the adaptation occurred soon after the start of the deficiency treatment and was upheld after 4 weeks or if methylation slowly changed. The reduction in methylation, especially in the CG context, was much more pronounced in -N than in -P samples. 98% of CG and 92% of CHG DMRs were lower methylated in -N than in the control, while Kou et al. (2011) found almost no change in the overall average methylation level in rice. With -P, a less pronounced loss in methylation with 77% of CG and 76% of CHG DMRs being hypomethylated, was found. This also contrasts the situation in rice, a species belonging to the same family of Poaceae, where Secco et al. (2015) found that 84% of DMRs under phosphorus-starvation conditions were hypermethylated. In *A. thaliana*, Yong-Villalobos et al. (2015) found that 86% of DMRs were hypermethylated after 16 days of phosphorus-deficiency treatment. Some of these differences in DNA methylation might be caused by the different time-points of sampling, different stress severity or by different criteria defining DMRs, but most probably also suggest species-specific differences.

Knowledge about DNA methylation adaptation to different nutrient-stresses and its influence on gene expression in maize is scant, as most research concerning these topics in plants has been done in the model *A. thaliana*. Since quite some time it is assumed that the possibly harmful activity of TEs is silenced by hypermethylation of transposons. Increasing evidence now suggests that changing methylation in transposons might also have an effect on gene expression regulation (Slotkin and Martienssen, 2007; Lisch and Bennetzen, 2011; Mirouze and Vitte, 2014). These mechanisms are difficult to investigate in *A. thaliana*, as it is a plant poor in transposons and methylation as well. Maize, on the other hand, has a giant genome, which is mainly composed of TEs. DNA and RNA sequencing enabled us to investigate parallel changes in the methylome and transcriptome of maize roots and revealed a highly nutrient-specific adaptation of mRNA transcripts and DNA methylation. The extent of transcriptional change was relatively similar between the two nutrient deficiency conditions, as well as the percentage of up- and down regulation. Certain well-known gene categories and individual marker genes, such as high affinity nitrate or phosphate transporters, were transcriptionally up-regulated in -N and -P, respectively. Many of these genes may represent nice markers for maize -N and -P deficiencies. Furthermore, a large set of overlapping genes was co-regulated under both deficiencies and probably indicates more unspecific stress (**Figure 3E**). We could show that many of the differentially methylated TEs seemed to have a gene expression regulatory effect on nearby genes. Secco et al. (2015) found that many hypermethylated TEs were located near induced genes in rice, while other teams suggested that hypermethylation of TEs leads to decreased gene expression of nearby genes in *A. thaliana* (Hollister and Gaut, 2009; Ahmed et al., 2011; Eichten et al., 2012). We found that most TEs were hypomethylated when compared to control and the closely nearby genes were both up- and down regulated in more or less equal parts. It is yet not possible to make a universally valid conclusion about the influence of hypermethylation in TEs on nearby genes that would apply to all plant species. Thus, our results challenge the often-made assumption that methylation represses transcription in plants. However, the theory that methylation in TEs has an influence on nearby gene expression is further supported by our data.

Surprisingly, no significant correlation between differential methylation in promoters and/or gene bodies and gene expression was present. 89% of all DMRs in -N and 95% in -P were located in TEs, which was even slightly more than expected considering the genome consisting to 85% of TEs. As a consequence, only a comparably small number of DMRs were located in genes and promoters. Of these, only between 6 and 19% in both symmetrical contexts and the two conditions were both differentially methylated and differentially expressed. These percentages are not higher than can be expected by coincidence.

RBBS has some advantages compared to whole genome bisulfite sequencing, as for example reducing time and especially cost needed to achieve proper sequencing depth by enriching the library for CG containing motifs. Nevertheless, it also has its drawbacks, the main of which for our experiments lies in the poor comparability of methylome and transcriptome. As we have the information of expression of all genes of the entire genome, but methylation information only for roughly 14% of the genome, it was not possible to evaluate the influence of methylation on gene expression for all genes, but only for a subset. This was made even harder by the fact that, for example, some genes are covered in full length by the reduced representation genome, while others were only partly covered, making detection of a DMR in these genes less likely. We tried to alleviate this difficulty in comparison by setting a cutoff for genes that were only covered partly by the reduced representation genome. Still, the results may be biased and it is possible that with reduced representation bisulfite sequencing, a significant correlation between DMRs in genes and expression escaped our analysis. However, our experiments clearly showed that maize roots adapt their DNA methylation in a highly nutrient-specific way. Additionally, we could provide further evidence that the methylation in TEs takes part in gene expression regulation of nearby genes.

## Conclusion

Though there are still a lot of open questions and things to learn about the role of DNA methylation in plants, it more and more crystallizes that the way DNA methylation adapts in different plants is strongly dependent on which plant species is concerned. To our knowledge, the impact of the deficiency of different essential nutrients on the methylome of maize plants has not been directly compared before and therefore this work provides a valuable basis for further research to overcome the gap between model plants like Arabidopsis, on which most experiments are still done, and plants which play a tremendous role in agriculture. The high adaptability of DNA methylation showed that it is highly dynamic, as a response to essential nutrient deficiencies. The exact roles for the plant, however, remain partially obscure.

It was often assumed that methylation is involved in gene expression and/or TE regulation, but general conclusions on repressive or activating functions of DNA methylations are not supported by our data. DNA methylation is definitely not the only epigenetic mechanism to influence gene expression. Only a detailed histone methylation and acetylation code, for example, in parallel with the methylome, is probably required to unravel the function in gene regulation by DNA methylation. Besides, the more different plant species are looked at and investigated, the clearer the picture will get concerning species- and tissue-specific influences. Finally, DNA methylation might primarily have other functions than regulation of gene expression as well, as for example protecting the DNA from harmful TE activity. All in all, this work provides basic information that will inspire further investigations about the dynamics and function of DNA methylation, which is likely valuable for plant breeding, crop protection and evolutionary studies.

## Notes

The raw data were submitted to NCBI and are available under the following code: SRP135301.

## Author Contributions

SM and UL contributed to the conception and design of the study. SM performed the statistical analysis and wrote the first draft of the manuscript. UL wrote the sections of the manuscript. All authors contributed to the manuscript revision, read and approved the submitted version.

## Conflict of Interest Statement

The authors declare that the research was conducted in the absence of any commercial or financial relationships that could be construed as a potential conflict of interest.
