# Supplementary material for: Massive Loss of DNA Methylation in Nitrogen-, but Not in Phosphorus-Deficient Zea mays Roots Is Poorly Correlated With Gene Expression Differences
Source: Front Plant Sci. 2018 Apr 19;9:497. doi: 10.3389/fpls.2018.00497 (PMC5917015; doi:10.3389/fpls.2018.00497)
Supplement: Supplementary file 1 [file Image_1.PDF]

Supplementary Material

Massive Loss of DNA Methylation in Nitrogen-, but not in Phosphorus-deficient *Zea mays* Roots is poorly correlated with Gene Expression Differences

Svenja Mager, Uwe Ludewig

Correspondence: Uwe Ludewig, University of Hohenheim, Germany
u.ludewig@uni-hohenheim.de

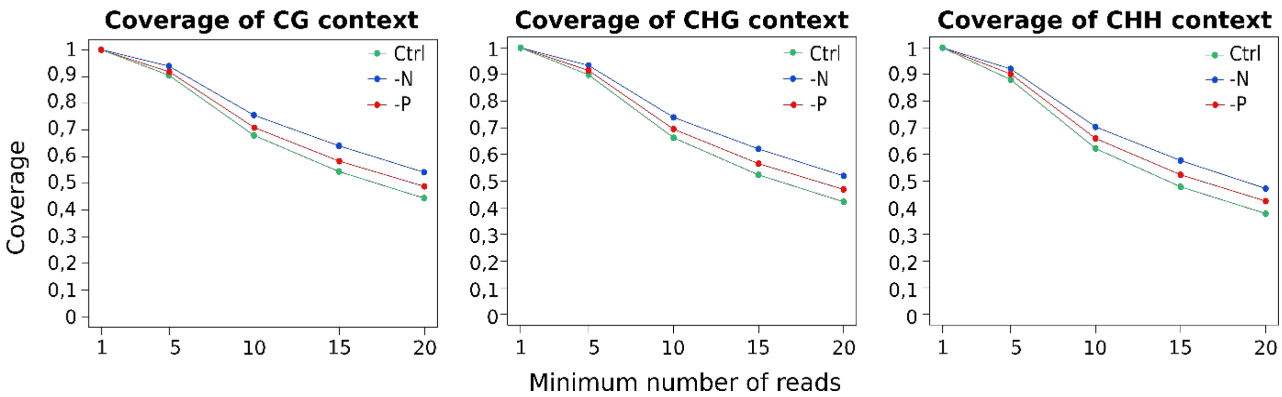

Supplementary Figure 1: Coverage of Cytosines in CG, CHG and CHH contexts in Control, -N and –P samples.

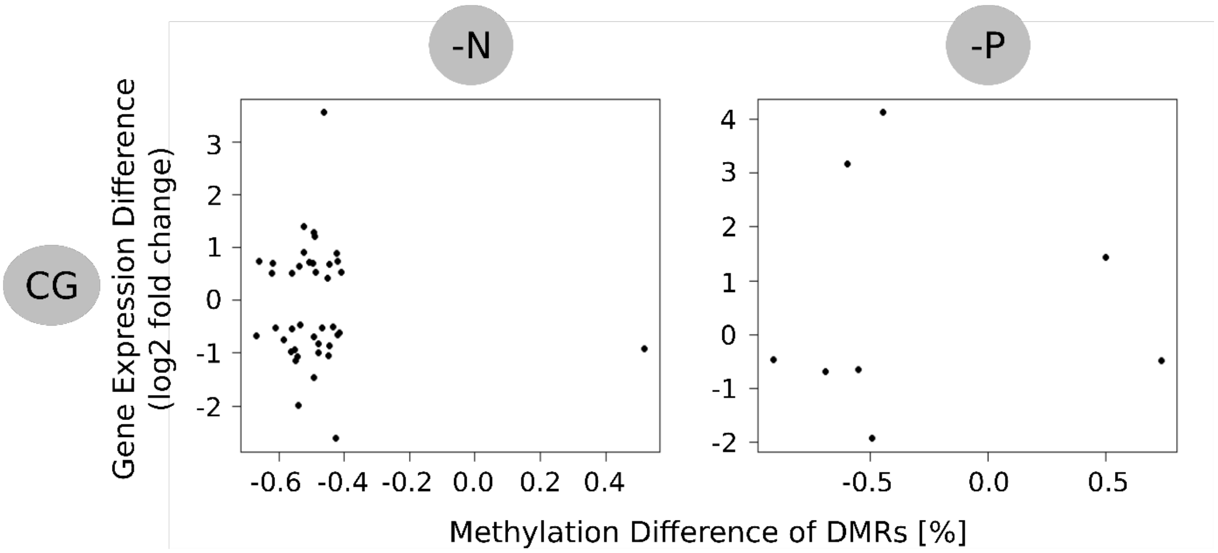

Supplementary Figure 2: Genes, which are differentially expressed and differentially methylated in CG context.

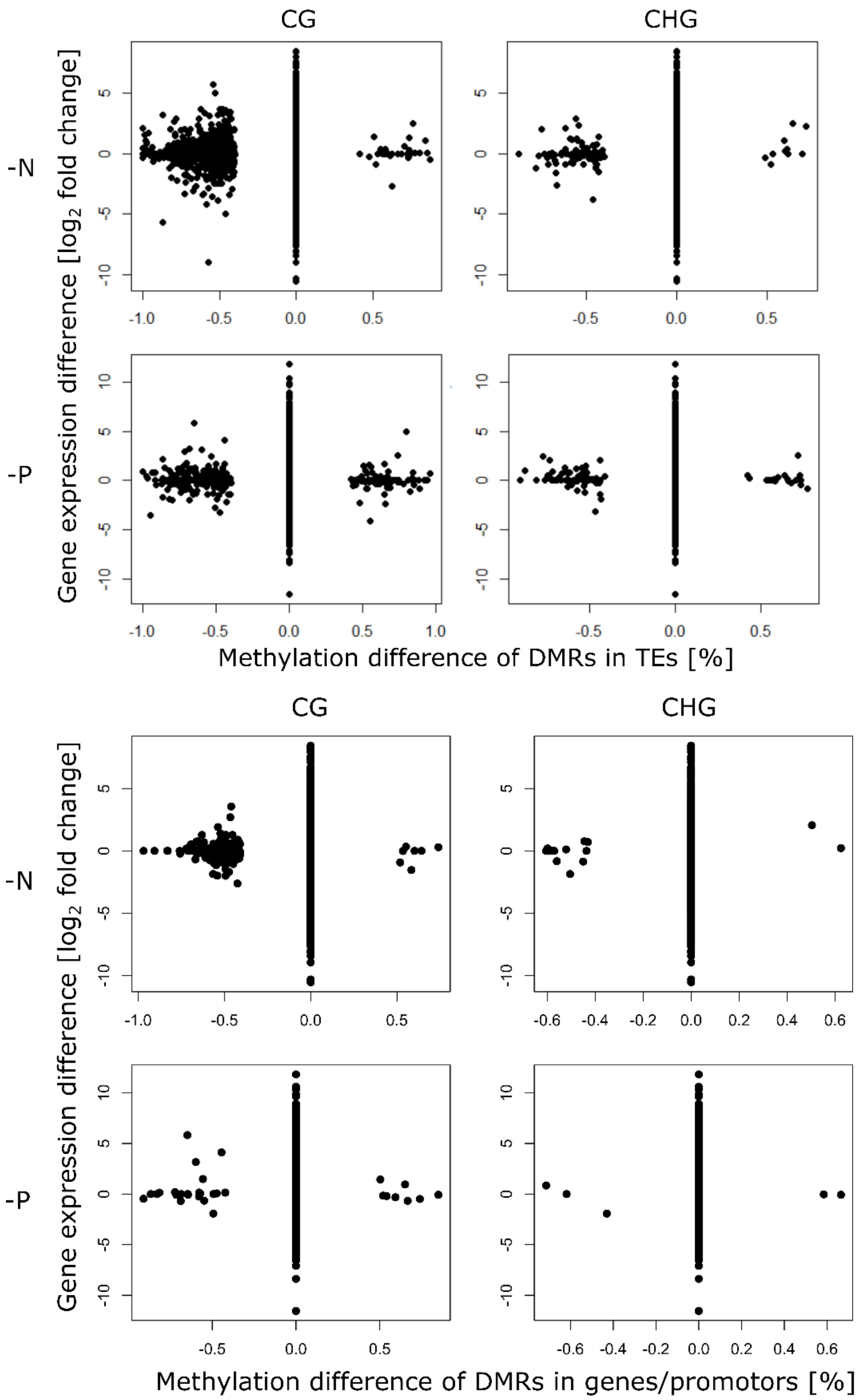

Supplementary Figure 3: (A) Genes of which some are differentially expressed and some contain a DMR in the gene body/promotor. **(B)** Genes of which some are differentially expressed and some contain a nearby differentially methylated TE.

Supplementary Table 1: Alignment output of RRBS libraries. Values are averaged among replicates and shown in millions.

	Control	- N	- P
Raw BS-read pairs	35.40	37.00	35.90
Multiple hits reads	0.13	0.13	0.13
Unmapped read pairs	18.20	19.00	18.38
Uniquely aligned read pairs	17.23	18.02	17.48
**Mappability**	**48.62%**	**48.70%**	**48.80%**

Supplementary Table 2: DMR count with DMRs being ≥ 10% differentially methylated.

	-N	-P
**CG**	5933	1290
**CHG**	1301	310
**CHH**	6	2

Supplementary Table 3: Alignment output of RNA-Sequencing. Values are averaged among replicates and shown in millions.

	Control	- N	- P
Paired reads	27.99	27.94	27.73
Unaligned reads	2.30	2.70	2.27
Multiply aligned reads	2.70	2.49	2.57
Uniquely aligned reads	22.99	22.74	22.89
**Alignement rate**	**91.78%**	**90.34%**	**91.81%**
